# Accelerated biological aging as a potential mediator mediates the relationship between metabolic syndrome and the risk of psoriasis: a prospective analysis from the UK biobank

**DOI:** 10.3389/fimmu.2025.1620027

**Published:** 2025-08-11

**Authors:** Rongqian Tian, Shaona Qiu, Jinrong Zhang, Ming Chen, Hai Yu, Waichi Lau, Jun Lyu, Liehua Deng

**Affiliations:** ^1^ Department of Dermatology, The First Affiliated Hospital of Jinan University & Jinan University Institute of Dermatology, Guangzhou, China; ^2^ Jinan University Institute of Dermatology, Guangzhou, China; ^3^ Department of Dermatology, The Fifth Affiliated Hospital of Jinan University, Heyuan, China; ^4^ Department of Basic Medical Sciences and Public Health, Jinan University, Guangzhou, China; ^5^ Department of Clinical Research, The First Affiliated Hospital of Jinan University, Guangzhou, China; ^6^ Guangdong Provincial Key Laboratory of Traditional Chinese Medicine Informatization, Guangzhou, China

**Keywords:** metabolic syndrome, psoriasis, genetic susceptibility, biological aging, mediation analysis

## Abstract

**Background:**

Increasing evidence suggests that metabolic syndrome (MetS) may contribute to the development of psoriasis. However, the mediating role of accelerated aging in this association remains unclear.

**Methods:**

This study utilized data from 319,263 participants in the UK Biobank. Cox proportional hazards models were used to assess the associations between MetS, genetic predisposition, and psoriasis risk. Mediation analysis examined the role of accelerated aging (PhenoAgeAccel) in the relationship between MetS, its components, and psoriasis.

**Results:**

MetS was associated with a 30% increased risk of psoriasis (HR: 1.30; 95% CI: 1.20–1.40). Among its components, abdominal obesity, low HDL cholesterol, high triglycerides, and hyperglycemia were each independently linked to higher risk. Individuals with both MetS and high genetic susceptibility had a substantially increased risk (HR: 2.93; 95% CI: 2.51–3.43). PhenoAgeAccel significantly mediated 28.8% of the MetS–psoriasis association.

**Conclusions:**

MetS and its components play a key role in psoriasis development, especially in genetically susceptible individuals. Accelerated aging may partially explain this link, suggesting a potential biological pathway and underscoring the importance of early MetS identification.

## Introduction

Psoriasis is a frequently occurring, chronic, and recurrent inflammatory skin condition characterized by erythematous plaques, scaling, and thickened lesions (1). The multifactorial pathogenesis of psoriasis is associated with abnormal keratinocyte proliferation driven by dysregulated immune responses (2, 3). Approximately 2% to 3% of the global population is affected, and both genders are at risk (4). Besides psoriasis’ prevalence and chronic state, it can result in considerable cosmetic disfigurement and disability (5, 6). These physical impacts often contribute to social isolation, psychological distress, and an increased risk of mental health disorders, including depression (7, 8).

Metabolic syndrome (MetS), a prevalent metabolic disorder, is characterized by a set of interrelated clinical manifestations, including central obesity, hypertension, hyperglycemia, and dyslipidemia, like elevated triglycerides and low high-density lipoprotein cholesterol levels (7, 9, 10). Studies have pointed out that the coexistence of these conditions leads to a twofold increase in cardiovascular disease risk and a fivefold increase in diabetes risk (11). Recent decades have witnessed a rise in the global prevalence of MetS, affecting around 25% of the population and accounting for 7% of total mortality (12). Transformations in modern living modes have put MetS as a major public health challenge across the world (13).

More attention is being paid to the relationship between psoriasis and MetS, and emerging evidence implies that MetS may stand as a novel risk factor for psoriasis (14, 15). The two conditions share several metabolic risk factors, including insulin resistance, obesity, hypertension, and dyslipidemia, which could collectively raise the risk of psoriasis through complex pathological mechanisms (16). Besides, chronic inflammation in psoriasis and metabolic abnormalities are closely associated, potentially contributing to disease progression via inflammatory pathways (17, 18).

Genetic factors significantly sway the onset of psoriasis, with the interaction between genetic susceptibility and environmental factors crucial for developing and spreading the disease (17). The reciprocal influence of MetS and genetic predisposition could hasten psoriasis development via immune or metabolic pathways. Further inquiry into these interactions is vital for enhancing the knowledge of psoriasis pathophysiology and formulating personalized treatment strategies.

Meanwhile, recent studies have demonstrated a close association between biological aging and metabolic syndrome (19). Biological aging has also been identified as a risk factor for the development of psoriasis (20, 21). However, few prospective studies have explored the potential mediating role of accelerated biological aging, limiting our understanding of the pathways through which this mechanism may influence psoriasis risk.

This study seeks to examine the associations of metabolic syndrome, its components, and genetic risk with psoriasis, and to assess the potential mediating role of accelerated biological aging in the MetS–psoriasis relationship.

## Methods

### Data source

**Figure 1:**
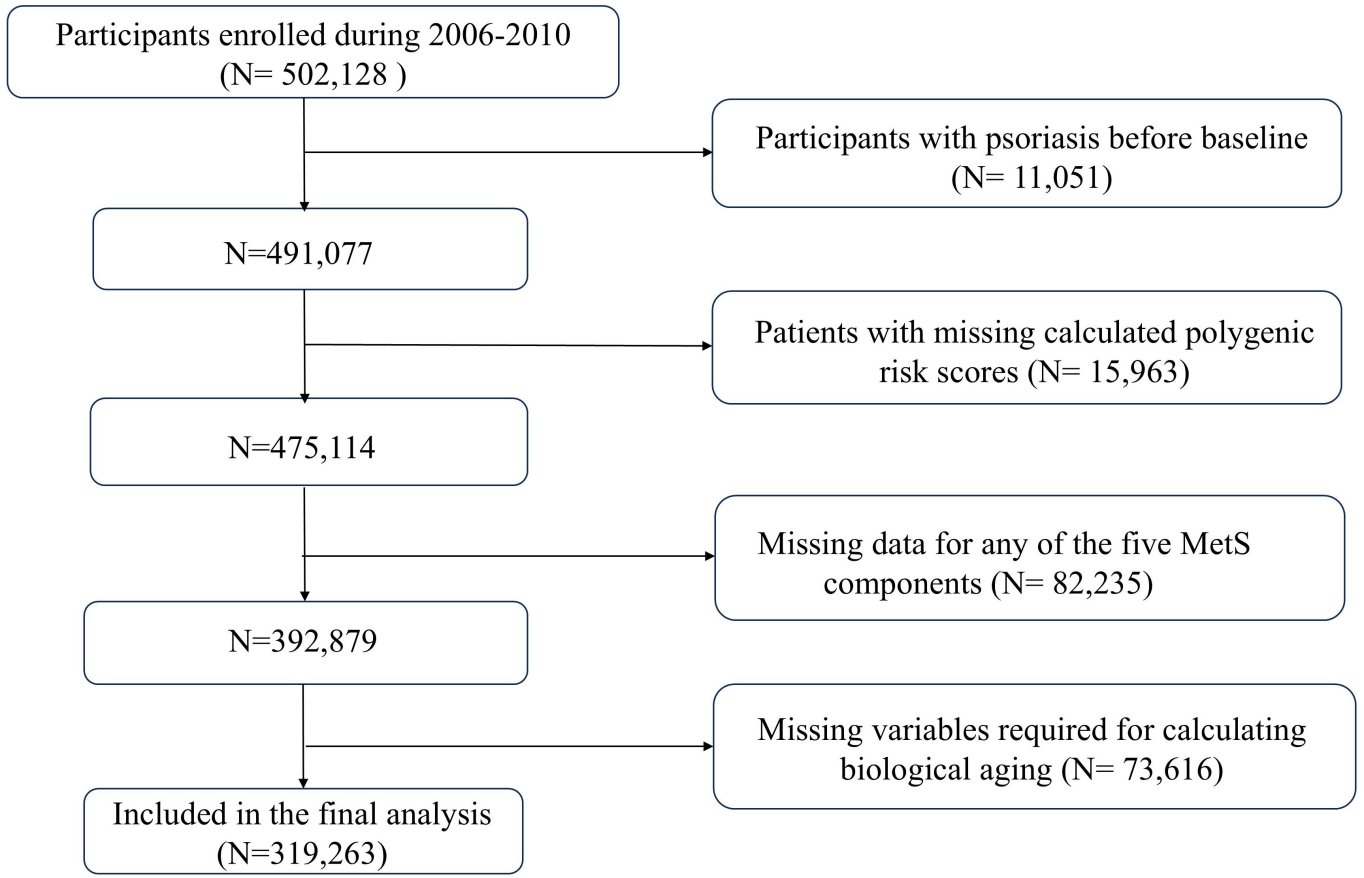
Selection process of the study population.

The data from the UK Biobank was applied in this prospective cohort study, which enrolled above 500,000 participants aged 37 to 73 years from April 2006 to December 2010, with follow-up concluding in July 2023. The participants were invited to take part in an assessment at one of 22 centers distributed across the UK, having sites in England, Scotland, and Wales. Detailed information regarding the UK Biobank was published previously (22). Participants were excluded under the condition that they (1) had psoriasis at baseline, (2) had data missing for any of the five components of metabolic syndrome or other relevant covariates, (3) lacked data on polygenic risk scores (PRS), or (4) had data missing for calculating biological aging. The sample choosing process is shown in **Figure 1**.

### Assessment of psoriasis

The diagnosis of psoriasis was primarily confirmed through hospital admission records obtained from the England Hospital Episode Statistics, Scotland Incidence Records, and the Wales Patient Event Database. Confirmation criteria included events recorded for the first time (field numbers 131742 and 131743), including primary care data, ICD codes in hospital inpatient records, ICD codes in death registration records, and self-reported health status codes validated by nurses after a doctor’s diagnosis. The follow-up period was defined as starting from the day each participant was evaluated up to July 6, 2023. For full-scale details regarding psoriasis assessment in the UK Biobank, access the UK Biobank’s official site (https://www.biobank.c.k/).

### Ascertainment of MetS

As per the 2009 standards developed by the American Heart Association, the National Heart, Lung, and Blood Institute, and the International Diabetes Federation, MetS is typified by having at least three of these elements: high blood pressure (systolic ≥130 mm Hg or diastolic ≥85 mm Hg); raised triglycerides (≥1.7 mmol/L); HDL cholesterol undergoes a reduction (<1 To men, the value reads 0 mmol/L, <1.3 mmol/L in women); escalated blood - sugar quantity, demonstrated by a raised HbA1c (≥42 mmol/mol [6.0%]); and elevated waist size.

### Genetic information

The research platform of the UK Biobank offers a uniform polygenic risk score (PRS), created by meta-analyzing data from various genome-wide association studies (GWAS). Prior research has enhanced the understanding of the PRS methodology and the standard PRS sets used in GWAS data (23). In brief, the UK Biobank employs standardized definitions for subgroups, diseases, and quantitative traits to assess PRS consistently. The PRS algorithm is based on a meta-analysis of specific traits, utilizing the Bastion method to integrate data across diverse ancestral populations and related traits. A higher PRS reflects increased genetic susceptibility to the disease. For psoriasis (Field ID, 26269), participants were categorized into low (quintile 1), medium (quintiles 2-4), and high (quintile 5) genetic risk categories according to the PRS quintiles (24).

### Covariates

Baseline sociodemographic and lifestyle data were collected via a touchscreen questionnaire. Sociodemographic variables comprised age (< 60 vs ≥ 60 years), race (white vs non-white), sex, Townsend Deprivation Index (divided into five quintiles), education level (degree, professional qualification, or secondary school), and household income (< £18,000, £18,000-£30,999, £31,000-£51,999, £52,000-£100,000, or > £100,000). Lifestyle factors included elements of smoking status (never, former, or current), alcohol consumption habit (never, former, or current), fasting time length (1–6 hours), activity (low, medium, high), and self-reported history of cardiovascular disease (CAD). A no-report category was included for participants who were uncertain or declined to respond.

### Phenotypic age and PhenoAgeAccel calculation

Biological aging in this study was assessed using the PhenoAge algorithm, which estimates all-cause mortality risk based on a Gompertz proportional hazards model incorporating chronological age and nine clinical biomarkers: albumin, alkaline phosphatase, creatinine, C-reactive protein, glucose, mean corpuscular volume, red cell distribution width, white blood cell count, and lymphocyte percentage. These biomarkers were initially identified through a Cox penalized regression model using data from the NHANES III cohort (25). PhenoAge acceleration (PhenoAgeAccel), reflecting deviations in biological aging relative to chronological age, was calculated as the residual from regressing PhenoAge on chronological age (26). To address the skewed distribution of certain biomarkers, we applied percentile-based winsorization, setting values below the 1st percentile and above the 99th percentile to the corresponding cutoff points to minimize the influence of outliers (27). All calculations were performed using the “BioAge” package in R.

### Statistical analysis

Descriptive statistical methods were employed to contrast the fundamental traits of participants who had Metabolic Syndrome (MetS) with those who did not. Variables of a categorical nature are denoted in percentage terms, whereas continuous variables are shown as average values with standard deviations (SD). The comparison of continuous and categorical variables across groups utilized T-tests, Wilcoxon rank-sum tests, and Chi-square (χ2) tests. Responses or missing data, such as “do not know/prefer not to answer,” were classified. The duration of follow-up was determined from the date of participant enrollment until either psoriasis diagnosis, death, dropout, or the study’s conclusion. The link between MetS, its elements, genetic vulnerability, and the risk of psoriasis was assessed using a Cox regression model, resulting in the computation of hazard ratios (HRs). The Schoenfeld residual-based test was used to confirm no significant deviations from proportional hazards in all models. Model 1 was adjusted for age, gender, household income, education level, Townsend deprivation index, and race. In Model 2, we further adjusted for polygenic risk score, cardiovascular disease history, smoking status, alcohol consumption, and fasting duration. To examine the joint exposure effects of MetS and genetic susceptibility on psoriasis risk, participants were categorized into six groups based on genetic risk (low, medium, high) and MetS (yes/no). Multicollinearity was assessed, and no significant issues were found. Additionally, the P-value for the trend was computed by coding joint exposure as a continuous variable. Stratified analyses were performed by gender (male, female) and age (< 60 years and ≥ 60 years).

Mediation analysis was conducted using the “mediation” package in R. First, a linear regression model was fitted to assess the association between the exposure (metabolic syndrome and its components) and the mediator (standardized PhenoAgeAccel), adjusting for potential confounders including age, sex, ethnicity, education level, TDI, income, smoking status, alcohol consumption, CAD history, fasting time, and physical activity. Next, a logistic regression model was constructed to evaluate the association between the exposure and the outcome (psoriasis), with both the exposure and mediator included in the model, adjusting for the same set of covariates. The average causal mediation effect (ACME) and the average direct effect (ADE) were estimated using the “mediate” function, with 1,000 bootstrap simulations to calculate confidence intervals.

Multiple sensitivity tests were performed to evaluate the solidity of the results, such as (1) omitting participants who had less than two years of follow-up to reduce differential bias; (2) excluding patients with fasting times less than 3 hours to reduce potential bias from serum metabolic biomarkers; (3) excluding patients with missing data of MetS components and covariates. Each P-value was bidirectional, establishing statistical significance at P<0.05. Every analysis was performed utilizing RStudio version 4.3.3.

## Results

### Baseline characteristics

This study included 319,263 participants, of whom 231,006 (72.4%) did not have metabolic syndrome and 88,257 (27.6%) had metabolic syndrome. **Table 1** displays initial traits categorized according to MetS status. Compared to those without MetS, individuals with a MetS diagnosis were more likely to be older, male, of non-white background, have lower levels of education, live in socioeconomically disadvantaged areas, earn less income, have a history of smoking, abstain from alcohol or be former drinkers, have a medium activity and report a prior diagnosis of coronary artery disease. At baseline, patients with MetS also manifested higher prevalences of hypertension, hyperglycemia, elevated triglycerides, central obesity, and low HDL cholesterol.

**Table 1:** Baseline characteristics of the study population by MetS status.

Characteristics	Non MetS (*N*=319,263)	MetS (*N*=231,006)	Total (*N*=319,263)	*P-value*
Age				<0.001
60 and above	94,082 (40.7%)	45,837 (51.9%)	139,919 (43.8%)	
Under 60	136,924 (59.3%)	42,420 (48.1%)	179,344 (56.2%)	
Gender				<0.001
Female	126,790 (54.9%)	44,362 (50.3%)	171,152 (53.6%)	
Male	104,216 (45.1%)	43,895 (49.7%)	148,111 (46.4%)	
Ethnicity				<0.001
White	220,864 (95.6%)	83,160 (94.2%)	304,024 (95.2%)	
Non-white	7,292 (3.2%)	3,760 (4.3%)	11,052 (3.5%)	
Missing	2,850 (1.2%)	1,337 (1.5%)	4,187 (1.3%)	
Education				<0.001
Degree	81,420 (35.2%)	22,004 (24.9%)	103,424 (32.4%)	
Professional qualification	11,494 (5.0%)	5,092 (5.8%)	16,586 (5.2%)	
Secondary school	102,378 (44.3%)	39,831 (45.1%)	142,209 (44.5%)	
Missing	35,714 (15.5%)	21,330 (24.2%)	57,044 (17.9%)	
Household income (in GBP)				<0.001
Less than 18,000	49,248 (21.3%)	20,423 (23.1%)	69,671 (21.8%)	
18,000 to 30,999	54,145 (23.4%)	18,082 (20.5%)	72,227 (22.6%)	
31,000 to 51,999	44,924 (19.4%)	11,901 (13.5%)	56,825 (17.8%)	
52,000 to 100,00	12,598 (5.5%)	2,449 (2.8%)	15,047 (4.7%)	
Greater than 100,000	38,648 (16.7%)	21,157 (24.0%)	59,805 (18.7%)	
Missing	31,443 (13.6%)	14,245 (16.1%)	45,688 (14.3%)	
Townsend deprivation index, quintiles				<0.001
1 (least deprived)	95,117 (41.2%)	32,157 (36.4%)	127,274 (39.9%)	
2	85,182 (36.9%)	32,215 (36.5%)	117,397 (36.8%)	
3	35,680 (15.4%)	16,125 (18.3%)	51,805 (16.2%)	
4	13,453 (5.8%)	6,921 (7.8%)	20,374 (6.4%)	
5 (most deprived)	1,298 (0.6%)	728 (0.8%)	2,026 (0.6%)	
Missing	276 (0.1%)	111 (0.1%)	387 (0.1%)	
Smoking status				<0.001
Current	21,144 (9.2%)	10,090 (11.4%)	31,234 (9.8%)	
Previous	77,237 (33.4%)	33,621 (38.1%)	110,858 (34.7%)	
Never	131,675 (57.0%)	43,997 (49.9%)	175,672 (55.0%)	
Missing	950 (0.4%)	549 (0.6%)	1,499 (0.5%)	
Alcohol status				<0.001
Current	216,060 (93.5%)	79,137 (89.7%)	295,197 (92.5%)	
Previous	6,593 (2.9%)	3,877 (4.4%)	10,470 (3.3%)	
Never	7,949 (3.4%)	4,975 (5.6%)	12,924 (4.0%)	
Missing	404 (0.2%)	268 (0.3%)	672 (0.2%)	
CAD history				<0.001
CAD	9,614 (4.2%)	7,418 (8.4%)	17,032 (5.3%)	
Non-CAD	221,392 (95.8%)	80,839 (91.6%)	302,231 (94.7%)	
Activity				<0.001
Low	28,826 (12.5%)	15,313 (17.4%)	44,139 (13.8%)	
Medium	93,964 (40.7%)	32,384 (36.7%)	126,348 (39.6%)	
High	60,204 (26.1%)	17,625 (20.0%)	77,829 (24.4%)	
Missing	48,012 (20.8%)	22,935 (26.0%)	70,947 (22.2%)	
Fasting time				<0.001
≤1	10,886 (4.7%)	4,102 (4.6%)	14,988 (4.7%)	
2	48,708 (21.1%)	17,475 (19.8%)	66,183 (20.7%)	
3	67,832 (29.4%)	25,528 (28.9%)	93,360 (29.2%)	
4	50,135 (21.7%)	20,341 (23.0%)	70,476 (22.1%)	
5	27,734 (12.0%)	11,083 (12.6%)	38,817 (12.2%)	
≥6	25,711 (11.1%)	9,728 (11.0%)	35,439 (11.1%)	
Elevated blood pressure				
SBP	137.4 ± 19.7	146.8 ± 17.5	140.0 ± 19.5	<0.001
DBP	81.0 ± 10.5	86.2 ± 10.0	82.5 ± 10.6	<0.001
Elevated HbA1c	5.3 ± 0.4	5.7 ± 0.6	5.4 ± 0.5	<0.001
Reduced HDL cholesterol	1.5 ± 0.4	1.2 ± 0.3	1.5 ± 0.4	<0.001
Elevated waist circumference	86.2 ± 11.2	100.9 ± 11.7	90.2 ± 13.1	<0.001
Hypertriglyceridemia	1.5 ± 0.8	2.5 ± 1.1	1.8 ± 1.0	<0.001
Albumin	45.4 ± 2.4	45.1 ± 2.4	45.3 ± 2.4	<0.001
Lymphocyte percentage	29.0 ± 6.7	29.2 ± 6.6	29.0 ± 6.6	<0.001
Mean cell volume	91.5 ± 3.8	90.5 ± 3.9	91.2 ± 3.8	<0.001
Glucose	4.9 ± 0.6	5.3 ± 1.0	5.0 ± 0.7	<0.001
Red cell distribution width	13.4 ± 0.7	13.5 ± 0.7	13.4 ± 0.7	<0.001
Creatinine	71.5 ± 13.1	73.1 ± 13.8	72.0 ± 13.3	<0.001
C-reactive protein	1.8 ± 2.3	3.2 ± 3.1	2.2 ± 2.6	<0.001
Alkaline phosphatase	80.5 ± 20.3	87.5 ± 21.1	82.5 ± 20.8	<0.001
White blood cell count	6.6 ± 1.5	7.3 ± 1.6	6.8 ± 1.5	<0.001

HbA1c, hemoglobin A1c; HDL, high-density lipoprotein; MetS, metabolic syndrome; SD, standard deviation; GBP, British pound sterling; CAD, cardiovascular disease; SBP, Systolic blood pressure; DBP, Diastolic blood pressure.

### MetS status, PRS, and risk of incident psoriasis

Exploring the link between metabolic syndrome and the occurrence of psoriasis, we performed a multivariable-adjusted Cox regression analysis, controlling for demographic variables in Model 1. Results in **Table 2** reveal a notable link between metabolic syndrome and a heightened likelihood of psoriasis. Taking Model 1 as a base, Model 2 was additionally adapted for factors like polygenic risk score, smoking status, alcohol status, fasting time, activity, and history of CAD, according to previous findings (**Table 2**). People diagnosed with MetS showed a 30% increased likelihood of developing psoriasis in comparison to those free of the condition (HR 1.30; 95% CI, 1.20-1.40). Particularly speaking, the elements of metabolic syndrome—hypertriglyceridemia (HR 1.14 95% CI, 1.06-1.23), hyperglycemia (HR 1.19; 95% CI, 1.09-1.30), magnified waist size (HR 1.32; 95% CI, 1.23-1.42), and was accompanied by a drop in HDL cholesterol (HR 1.25; 95% CI, 1.15-1.36) — were respectively related to a greater possibility of psoriasis. Yet, there was no obvious link between hypertension (HR 1.03; 95% CI, 0.95-1.12) coupled with the risk of psoriasis. Besides, the likelihood of psoriasis grew in proportion to the number of metabolic abnormalities.

**Table 2:** Association between metabolic syndrome status and risk of psoriasis.

MetS-Related Variables	No. of Cases	No. of Person-Years	Adjusted Model 1^a^		Adjusted Model 2^b^	
MetS			HR (95%CI)	*P-value*	HR (95%CI)	*P-value*
No	2013	4732372	Reference		Reference	
Yes	1086	2570546	1.34(1.24, 1.44)	< 0.001	1.30(1.20, 1.40)	< 0.001
Number of MetS						
0	351	835295	Reference		Reference	
1	826	1913949	0.98(0.87, 1.12)	0.792	0.99(0.87, 1.12)	0.822
2	836	1983128	1.13(1.00, 1.29)	0.059	1.12(0.98, 1.27)	0.090
3	628	1483698	1.30(1.14, 1.49)	< 0.001	1.27(1.11, 1.46)	< 0.001
4	345	845682	1.50(1.28, 1.74)	< 0.001	1.44(1.23, 1.67)	< 0.001
5	113	241166	1.81(1.46, 2.25)	< 0.001	1.69(1.36, 2.11)	< 0.001
*P* for trend	<0.001					
MetS components						
Hypertriglyceridemia						< 0.001
No	1686	3961913	Reference		Reference	1686
Yes	1413	3341005	1.17(1.09, 1.26)	< 0.001	1.14(1.06, 1.23)	
Reduced HDL cholesterol						< 0.001
No	2374	5588213	Reference		Reference	
Yes	725	1714705	1.28(1.18, 1.39)	< 0.001	1.25(1.15, 1.36)	
Elevated waist circumference						< 0.001
No	1828	4253404	Reference		Reference	
Yes	1271	3049514	1.35(1.26, 1.45)	< 0.001	1.32(1.23, 1.42)	
Elevated HbA1c						< 0.001
No	2429	5760530	Reference		Reference	
Yes	670	1542388	1.27(1.16, 1.38)	< 0.001	1.19(1.09, 1.30)	
Elevated blood pressure						0.447
No	851	2030673	Reference		Reference	
Yes	2248	5272245	1.01(0.93, 1.09)	0.898	1.03(0.95, 1.12)	

HbA1c, hemoglobin A1c; HDL, high-density lipoprotein; MetS, metabolic syndrome; HR, hazard ratio; 95% CI, 95% confidence interval.

a Adjusted for age, gender, ethnicity, education, Townsend deprivation index, and household income.

b Additionally adjusted for polygenic risk score, smoking status, drinking status, cardiovascular disease, activity, and fasting time based on adjusted Model 1.

**Table 3** shows how genetic risk affects psoriasis development. We measured genetic risk using polygenic risk scores. After adjustment, higher genetic risk groups had progressively higher psoriasis risk. The medium-risk group had 39% higher risk than the low-risk group (HR=1.39; 95%CI=1.25-1.54; p<0.001). The high-risk group had 2.22 times higher risk than the low-risk group (HR=2.22; 95%CI=1.98-2.49; p<0.001). The risk increased significantly with higher genetic risk scores (p-trend<0.001).

**Table 3:** Association between genetic predisposition and risk of psoriasis.

PRS	Total (*N*=392881)	Incident Psoriasis (*N*=3899)	No psoriasis (*N*=388982)	Adjusted Model 1^a^		Adjusted Model 2^b^	
				HR (95%CI)	*P-value*	HR (95%CI)	*P-value*
Low risk	63853	419	63434	Reference		Reference	
Middle risk	191557	1752	189805	1.39 (1.25, 1.55)	< 0.001	1.39 (1.25, 1.54)	< 0.001
High risk	63853	928	62925	2.23 (1.99, 2.5)	< 0.001	2.22 (1.98, 2.49)	< 0.001
*P* for trend					< 0.001		< 0.001

PRS, polygenic risk score; HR, hazard ratio; 95% CI, 95% confidence interval.

a Adjusted for age, gender, ethnicity, education, Townsend deprivation index, and household income.

b Additionally adjusted for smoking status, drinking status, cardiovascular disease, activity, and fasting time based on adjusted Model 1.

### Effect of interaction between MetS status and PRS on psoriasis risk

According to the data presented in **Table 4**, the link between metabolic syndrome and the risk of psoriasis varies based on genetic vulnerability levels. In individuals with moderate genetic susceptibility, those with metabolic syndrome had a 27% increased risk of psoriasis (HR, 1.27; 95% CI, 1.15-1.41; P < 0.001). In individuals with high genetic susceptibility, the association was even stronger, with a 41% increased risk (HR, 1.41; 95% CI, 1.23-1.62; P < 0.001). No significant interaction between metabolic syndrome and genetic susceptibility was observed regarding psoriasis risk (P = 0.143). Further analysis of psoriasis risk revealed that all components of metabolic syndrome– such as increased waist circumference, lower HDL cholesterol, higher fasting blood glucose, and higher triglyceride levels– are associated with increased psoriasis risk. This association was more pronounced among individuals with high genetic susceptibility. However, elevated blood pressure did not demonstrate a significant association with psoriasis risk. Additionally, a significant interaction was found between elevated triglycerides and genetic risk (P = 0.021).

**Table 4:** Association between metabolic syndrome status and risk of psoriasis according to genetic predisposition.

MetS		Low PRS status		Middle PRS status		High PRS status	
		No	Yes	No	Yes	No	Yes
No. of cases		46336	17517	138570	52987	46100	17753
No. of incident psoriasis		287	132	1147	605	579	349
Person-years		677486	328504	2679800	1418800	1375086	823242
Adjusted Model 1^a^	HR (95% CI)	Reference	1.17 (0.95, 1.45)	Reference	1.31 (1.19, 1.45)	Reference	1.46 (1.28, 1.68)
	*P-value*		0.132		<0.001		<0.001
Adjusted Model 2^b^	HR (95% CI)	Reference	1.16 (0.94, 1.44)	Reference	1.27 (1.15, 1.41)	Reference	1.41 (1.23, 1.62)
	*P-value*		0.164		<0.001		<0.001
*P* for interaction ^c^	0.143						
Elevated waist circumference							
No. of cases		42815	21038	128173	63384	42486	21367
No. of incident psoriasis		261	158	1044	708	523	405
Person-years		610401	395589	2440094	1658506	1202909	995419
Adjusted Model 1^a^	HR (95% CI)	Reference	1.21 (0.99, 1.48)	Reference	1.33 (1.21, 1.46)	Reference	1.46 (1.28, 1.67)
	*P-value*		0.064		<0.001		<0.001
Adjusted Model 2^b^	HR (95% CI)	Reference	1.20 (0.98, 1.47)	Reference	1.30 (1.18, 1.43)	Reference	1.43 (1.26, 1.64)
	*P-value*		0.08		<0.001		<0.001
*P* for interaction ^c^	0.160						
Reduced HDL cholesterol							
No. of cases		51734	12119	154718	36839	51807	12046
No. of incident psoriasis		335	84	1340	412	699	229
Person-years		786988	219002	3165541	933059	1635684	562644
Adjusted Model 1^a^	HR (95% CI)	Reference	1.09 (0.85, 1.38)	Reference	1.29 (1.15, 1.44)	Reference	1.38 (1.18, 1.6)
	*P-value*		0.506		<0.001		<0.001
Adjusted Model 2^b^	HR (95% CI)	Reference	1.08 (0.85, 1.38)	Reference	1.25 (1.12, 1.4)	Reference	1.31 (1.13, 1.53)
	*P-value*		0.521		<0.001		<0.001
*P* for interaction ^c^	0.238						
Elevated blood pressure							
No. of cases		18453	45400	55668	135889	18816	45037
No. of incident psoriasis		117	302	471	1281	263	665
Person-years		273730	732260	1104714	2993886	652229	1546099
Adjusted Model 1^a^	HR (95% CI)	Reference	0.97 (0.78, 1.21)	Reference	1.03 (0.92, 1.15)	Reference	0.98 (0.84, 1.13)
	*P-value*		0.773		0.574		0.752
Adjusted Model 2^b^	HR (95% CI)	Reference	0.98 (0.78, 1.22)	Reference	1.06 (0.95, 1.18)	Reference	1.01 (0.87, 1.17)
	*P-value*		0.855		0.318		0.909
*P* for interaction ^c^	0.902						
Elevated HbA1c							
No. of cases		53410	10443	158613	32944	52401	11452
No. of incident psoriasis		338	81	1376	376	715	213
Person-years		812468	193522	3246622	851978	1701440	496888
Adjusted Model 1^a^	HR (95% CI)	Reference	1.17 (0.91, 1.49)	Reference	1.26 (1.12, 1.41)	Reference	1.30 (1.11, 1.53)
	*P-value*		0.227		<0.001		0.001
Adjusted Model 2^b^	HR (95% CI)	Reference	1.13 (0.88, 1.46)	Reference	1.19 (1.06, 1.34)	Reference	1.22 (1.04, 1.43)
	*P-value*		0.332		0.004		0.017
*P* for interaction ^c^	0.905						
Hypertriglyceridemia							
No. of cases		38128	25725	114122	77435	37794	26059
No. of incident psoriasis		244	175	964	788	478	450
Person-years		577113	428877	2250434	1848166	1134366	1063962
Adjusted Model 1^a^	HR (95% CI)	Reference	1.03 (0.85, 1.26)	Reference	1.15 (1.04, 1.26)	Reference	1.27 (1.12, 1.45)
	*P-value*		0.765		0.005		<0.001
Adjusted Model 2^b^	HR (95% CI)	Reference	1.02 (0.84, 1.25)	Reference	1.12 (1.02, 1.24)	Reference	1.25 (1.09, 1.43)
	*P-value*		0.83		0.018		<0.001
*P* for interaction ^c^	0.021						

HbA1c, hemoglobin A1c; HDL, high-density lipoprotein; MetS, metabolic syndrome; PRS, polygenic risk score; HR, hazard ratio; 95% CI, 95% confidence interval.

a Adjusted for age, gender, ethnicity, education, Townsend deprivation index, and household income.

b Additionally for smoking status, drinking status, cardiovascular disease, activity, and fasting time based on adjusted Model 1.

c for interaction was calculated by involving the cross-product term of MetS status and genetic risk score in the fully adjusted Cox proportional hazards regression model.

### Association between MetS status and risk of psoriasis according to PRS

**Figure 2:**
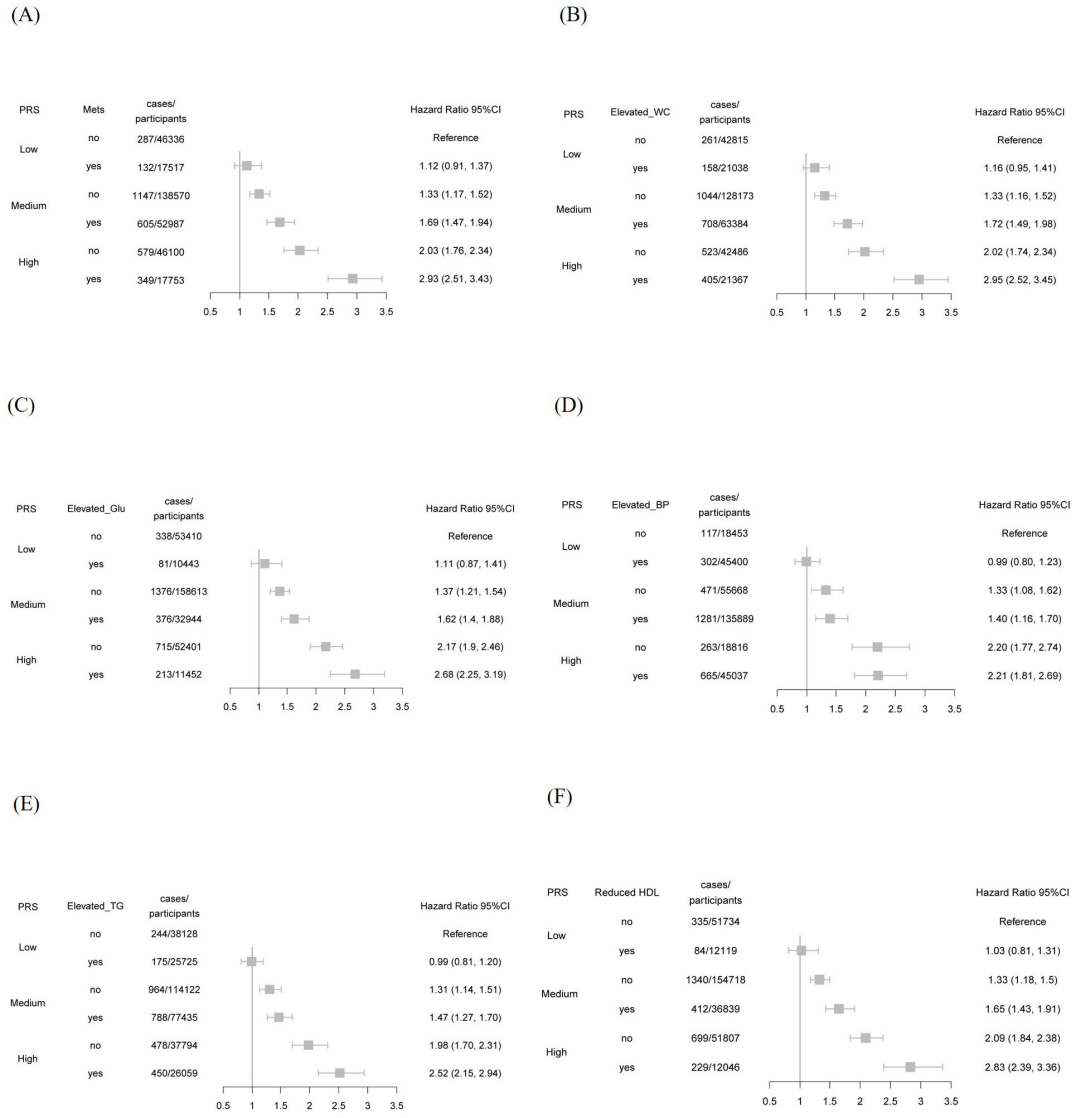
The risk of developing psoriasis was assessed according to metabolic syndrome (MetS) status and genetic susceptibility. **(A)** Metabolic syndrome (MetS); **(B)** Elevated waist circumference (WC); **(C)** Elevated glucose (Glu); **(D)** Elevated blood pressure (BP); **(E)** Elevated triglyceride (TG); **(F)** Reduced high-density lipoprotein (HDL).

In **Figure 2**, we examined the joint association of metabolic syndrome status and PRS with the risk of psoriasis development. In the fully adjusted model, participants with both metabolic syndrome and high genetic susceptibility exhibited a significantly elevated risk of psoriasis compared to those without metabolic syndrome and with low genetic susceptibility, with an HR of 2.93 (95% CI, 2.51-3.43). When evaluating specific metabolic components, the HRs were as follows: 2.68 (95% CI, 2.25-3.19) for elevated fasting glucose, 2.83 (95% CI, 2.39-3.36) for reduced high-density lipoprotein cholesterol, 2.52 (95% CI, 2.15-2.94) for elevated triglycerides, 2.21 (95% CI, 1.81-2.69) for elevated blood pressure, and 2.95 (95% CI, 2.52-3.45) for increased waist circumference. These findings suggest that the presence of components of metabolic syndrome, particularly associated with high genetic susceptibility, significantly increases the risk of developing psoriasis.

### Sensitivity analyses and subgroup

Sensitivity analyses were performed after excluding participants with missing MetS component and covariate data, less than 2 years of follow-up, or fasting times under 3 hours. As reported in **Supplementary File 1**, the presence of MetS and its components was significantly associated with an increased risk of psoriasis, and the dose-response relationship was significant with an increase in the number of MetS components (P represents trend < 0.001). Furthermore, **Supplementary File 2** revealed that the presence of MetS continued to be associated with a higher risk of psoriasis across different PRS categories. These results enhance the performance of MetS, its components, and the risk of psoriasis under varying analytical conditions.

The analysis categorized results based on age, gender, and genetic predisposition. Findings indicated that metabolic syndrome was linked to a 46% higher risk of psoriasis in individuals younger than 60 years (HR: 1.46, 95% CI: 1.31-1.62) and a 17% increase in those aged 60 and above (HR: 1.17, 95% CI: 1.05-1.30). Among participants with a high polygenic risk score, MetS further amplified the risk, with HRs of 2.48 (95% CI: 1.98-3.12) for individuals over 60 and 3.49 (95% CI: 2.81-4.34) for those under 60. Gender-based analysis showed that MetS raised psoriasis risk by 36% in women (HR: 1.36, 95% CI: 1.22-1.51) and 25% in men (HR: 1.25, 95% CI: 1.12-1.39). Additionally, individuals with both high PRS and MetS exhibited a more than threefold risk increase, with HRs of 2.93 (95% CI: 2.34-3.67) in women and 2.96 (95% CI: 2.37-3.70) in men. Additional findings are available in **Supplementary Files 3**-**6**.

### The mediating role of PhenoAgeAccel in the association between MetS and psoriasis risk

**Figure 3:**
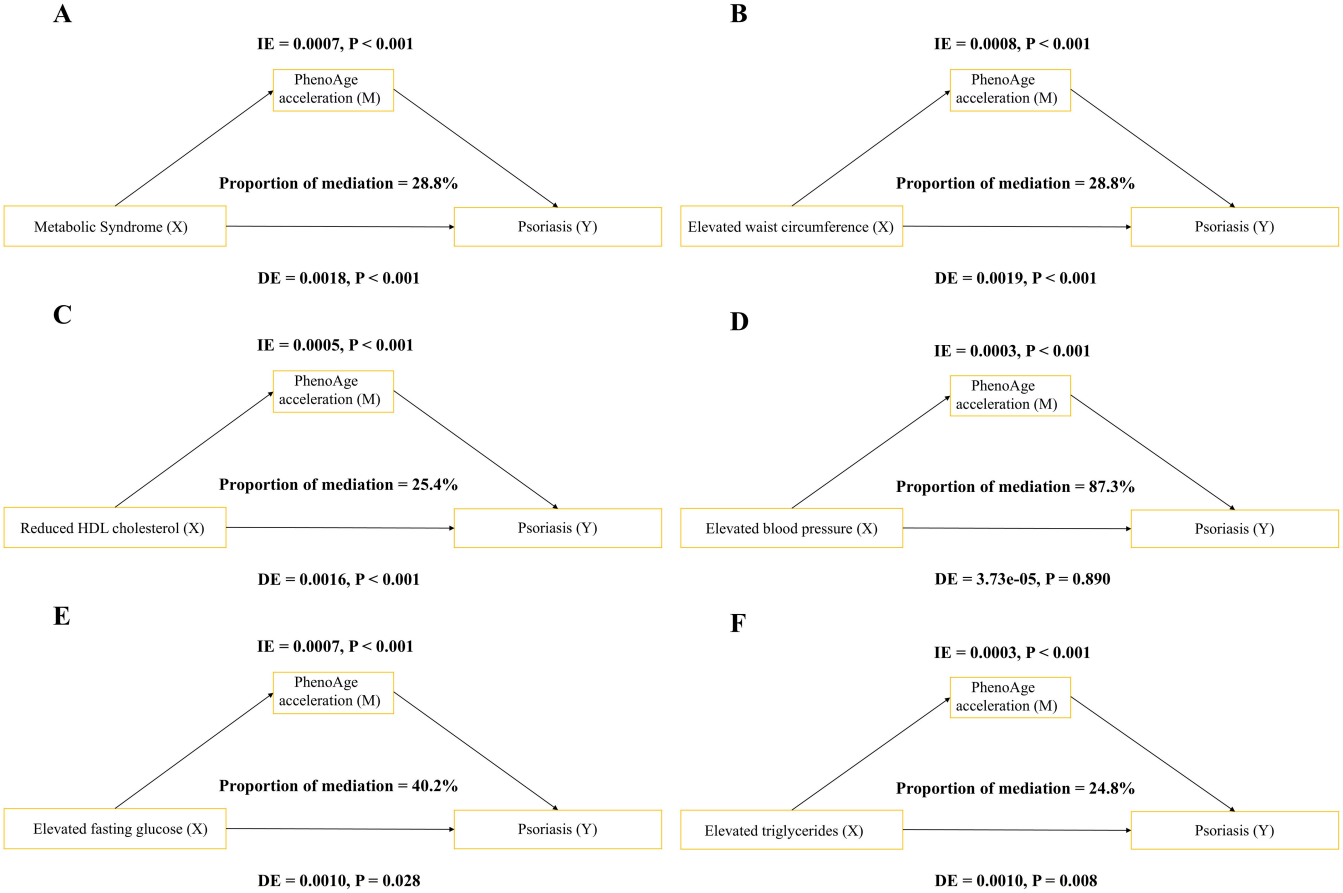
PhenoAge acceleration mediated the association between metabolic syndrome (MetS) components and the occurrence of psoriasis. **(A)** Metabolic syndrome (MetS); **(B)** Elevated waist circumference (WC); **(C)** Reduced high-density lipoprotein (HDL); **(D)** Elevated blood pressure (BP); **(E)** Elevated fasting glucose (Glu); **(F)** Elevated triglyceride (TG).

Mediation analysis indicated that PhenoAgeAccel acted as a mediator between metabolic syndrome and the risk of psoriasis (IE = 0.0007; DE = 0.0018; proportion mediated = 28.8%; P < 0.001) (**Figure 3A**).

When examining individual components of metabolic syndrome, PhenoAge acceleration mediated 28.8% of the association between increased waist circumference and psoriasis risk (IE = 0.0008; DE = 0.0019; P < 0.001) (**Figure 3B**) and 25.4% of the association between reduced HDL cholesterol and psoriasis risk (IE = 0.0005; DE = 0.0016) (**Figure 3C**). Additionally, PhenoAgeAccel mediated 40.2% of the association between elevated fasting glucose and psoriasis risk (IE = 0.0007; DE = 0.0010) (**Figure 3E**) and 24.8% of the association between elevated triglycerides and psoriasis risk (IE = 0.0003; DE = 0.0010) (**Figure 3F**). In the elevated blood pressure group, although the indirect effect was significant (IE = 0.0003; P < 0.001), the direct effect and the proportion mediated were not significant (**Figure 3D**).

To further verify the mediating role of PhenoAgeAccel, several sensitivity analyses were conducted, and the results remained largely consistent with the primary findings (**Supplementary Figures 1**-**3**).

## Discussion

In this extensive prospective cohort study involving 319,263 UK Biobank participants, MetS was linked to a 30% higher risk of developing psoriasis. Genetic susceptibility, assessed using a polygenic risk score, further modified this association, with MetS significantly amplifying psoriasis risk among individuals with high genetic predisposition. Among the components of MetS, elevated fasting glucose, increased triglyceride levels, larger waist circumference, and lower high-density lipoprotein cholesterol were each linked to a higher risk of psoriasis. In contrast, no significant association was found for elevated blood pressure. Mediation analysis showed that biological aging significantly mediated the association between metabolic syndrome and the risk of psoriasis, with a mediation proportion of 28.8%. To our knowledge, this is the first large-scale cohort study to explore the relationship between metabolic syndrome, genetic susceptibility, and the risk of psoriasis, while also examining the mediating role of accelerated biological aging.

For instance, a Norwegian cohort study reported that MetS was associated with a higher risk of psoriasis (RR: 1.66, 95% CI: 1.30-2.14) (28). However, most studies have primarily examined the potential mechanisms underlying the development of metabolic disorders following psoriasis rather than examining whether MetS precedes psoriasis onset. A meta-analysis reported a higher incidence of psoriasis compared to the general population (RR, 2.26; CI, 95%, 1.70-3.01) (29). Similarly, a cross-sectional study identified an increased prevalence of MetS in individuals with psoriasis (30). Findings from the present cohort study further support these associations, showing a significant 30% increased risk of psoriasis.

Psoriasis is a chronic inflammatory condition characterized by systemic inflammation (1), with elevated levels of proinflammatory cytokines, including interleukin-1β, interleukin-6, and tumor necrosis factor α. These inflammatory mediators promote oxidative damage and impair insulin signaling throughout the body, which are fundamental characteristics of metabolic syndrome (31, 32). Metabolic abnormalities may further exacerbate inflammation, promoting the onset and progression of psoriasis (1, 33).

In this study, several components of MetS were linked with an increased risk of psoriasis. Increased waist circumference was positively associated with psoriasis, which is consistent with previous findings. An American prospective cohort study reported that larger waist circumference was associated with a higher risk of psoriasis (multivariable relative risk [RR], highest vs lowest tertile, 1.50; 95% CI, 1.24-1.82) (34). This association may be attributed to abdominal fat accumulation, which promotes the secretion of proinflammatory cytokines such as tumor necrosis factor α, interleukin 6, interleukin 17, and interleukin 23 (35, 36). These cytokines are directly involved in psoriasis pathogenesis through the activation of Th17 and Th1 cells, promoting abnormal keratinocyte proliferation and cutaneous inflammation (37, 38).

Lower HDL cholesterol levels and higher triglyceride levels also increase the risk of psoriasis. This finding is consistent with a cross-sectional analysis from the UK Biobank, which reported that HDL deficiency and elevated triglycerides were associated with 16.6% and 10.6% increased psoriasis risk, respectively (39). Additionally, an observational and Mendelian randomization analysis reported an association between elevated plasma triglycerides and psoriasis risk (40).

Elevated fasting glucose levels were linked with a 22% increased risk of psoriasis, consistent with findings from a prior cohort study (41). That study estimated that psoriasis patients accounted for approximately 125,650 additional new cases of type 2 diabetes annually worldwide compared with individuals without psoriasis. Hyperglycemia is known to promote systemic inflammation through the activation of multiple inflammatory pathways, including NF-κB and JAK-STAT, which are closely linked to psoriasis pathogenesis (42, 43).

Although previous studies have shown an increased risk of hypertension, particularly resistant hypertension, among patients with severe psoriasis (44), the research we conducted found no notable link between increased blood pressure and the risk of psoriasis.

Finally, a positive correlation was noted between the quantity of MetS elements and the risk of psoriasis (P for trend < 0.001). The results of this study offer a significant understanding of the specific MetS components that may contribute to psoriasis development and highlight the potential benefit of managing these metabolic factors to reduce psoriasis risk.

This study found that the combination of genetic factors and metabolic syndrome plays a significant role in the onset of psoriasis. Participants in the group with high PRS faced a risk of psoriasis that was 2.93 times higher than those in the low PRS group. As far as we are aware, this research is pioneering in assessing whether the link between metabolic syndrome and the risk of psoriasis differs based on genetic vulnerability.

An analysis segmented by age and gender revealed a notable link between MetS and a heightened risk of psoriasis among all subgroups. This correlation was more noticeable in people under 60 years old (HR, 1.46; 95% CI, 1.31-1.62), aligning with results from earlier research (45). The link between MetS and the risk of psoriasis was marginally more pronounced in women (HR, 1.36; 95% CI, 1.22-1.51) compared to men (HR, 1.25; 95% CI, 1.12-1.39). This difference may be related to hormonal fluctuations in women, linked to a rise in visceral fat build-up and higher levels of chronic inflammation, potentially contributing to greater metabolic risk (46–48). Additional sensitivity studies reinforced the strong link between MetS and the risk of psoriasis.

Mediation analysis revealed that accelerated biological aging significantly mediated the relationship between metabolic syndrome and psoriasis. Specifically, biological aging mediated the association between metabolic abnormalities, including increased waist circumference, reduced HDL cholesterol, elevated fasting glucose, and elevated triglycerides, and the risk of developing psoriasis. This suggests that biological aging is not only a consequence of metabolic syndrome but also plays a crucial role in driving the onset of psoriasis. A recent study has suggested that biological aging could be a potential risk factor for late-onset psoriasis (21). Moreover, accelerated biological aging may activate chronic low-grade inflammation pathways, particularly NF-κB and JAK-STAT signaling, leading to elevated systemic inflammation (49, 50). This indicates that biological aging may exacerbate metabolic abnormalities by influencing immune system function and promoting systemic inflammation, thereby contributing to the development of psoriasis. These findings not only provide new insights into the association between metabolic syndrome and psoriasis but also suggest that biological aging could be a potential intervention target for psoriasis, offering a novel approach for the prevention and treatment of the disease. The relationship between metabolic syndrome and biological aging may be bidirectional. Although the current study highlights the mediating role of biological aging in the association between metabolic syndrome and psoriasis, prior evidence suggests that metabolic dysfunction may itself accelerate biological aging through mechanisms involving oxidative stress, mitochondrial dysfunction, and chronic systemic inflammation (51–53). This raises the possibility of a feedback loop in which metabolic disturbances promote biological aging, which in turn enhances inflammatory processes that contribute to psoriasis pathogenesis. Further research using longitudinal and interventional study designs is warranted to clarify the directionality and mechanistic underpinnings of this relationship. The research presents multiple advantages. It included a large sample size, a prospective design, and independently obtained data on MetS, demonstrating a longitudinal association between metabolic syndrome and its components with the risk of psoriasis in real-life settings. The study also incorporated large-scale genetic risk score data, enabling extensive stratification of gene susceptibility and allowing for an evaluation of whether the association between MetS and psoriasis varies across different levels of genetic risk. This study has several limitations. First, the participants were predominantly middle-aged and older adults of European descent, which may limit the generalizability of the findings to other racial and ethnic populations. Second, the absence of clinical data, including Psoriasis Area and Severity Index scores (PASI) and treatment information, precluded assessment of associations between metabolic syndrome and disease severity. Third, the observational design limits causal inference, and residual confounding cannot be excluded. Longitudinal studies or Mendelian randomization approaches may help clarify causal relationships. Finally, psychological stress, which may influence metabolic and immune pathways, was not assessed. Future research should incorporate validated stress measures or biomarkers (e.g., cortisol) to better evaluate its potential role.

## Conclusion

In this large-scale prospective cohort study, metabolic syndrome was significantly associated with an increased risk of developing psoriasis, with the association being even stronger among individuals with higher genetic susceptibility. Specific components of MetS, including increased waist circumference, elevated fasting glucose, higher triglyceride levels, and reduced high-density lipoprotein cholesterol, were each independently linked to a greater risk of psoriasis. Mediation analysis further revealed that accelerated biological aging played a significant mediating role in the relationship between MetS and psoriasis risk, suggesting that biological aging is not merely a consequence of metabolic disturbances but also an important driver in the development of psoriasis. These findings enhance our understanding of the interplay between metabolic health, genetic predisposition, and psoriasis risk and highlight biological aging as a potential target for future prevention and intervention strategies against psoriasis.

## Data Availability

The raw data supporting the conclusions of this article will be made available by the authors, without undue reservation.
