# Supplementary material for: Accelerated biological aging as a potential mediator mediates the relationship between metabolic syndrome and the risk of psoriasis: a prospective analysis from the UK biobank
Source: Front Immunol. 2025 Aug 11;16:1620027. doi: 10.3389/fimmu.2025.1620027 (PMC12375602; doi:10.3389/fimmu.2025.1620027)
Supplement: Supplementary file 1 [file Supplementaryfile1.docx]

Supplemental File 1 Sensitivity analysis of the hazard ratio of metabolic syndrome (MetS) and its components to the risk of psoriasis

|  | Complete missing data of MetS components and covariates | | | | Excl. Participants with a follow-up <2 years | | | | Excl. Fasting time less than 3 hours | | | |
| --- | --- | --- | --- | --- | --- | --- | --- | --- | --- | --- | --- | --- |
|  | No. of Cases | Person-years | HR (95% CI) | *P-value* | No. of Cases | Person-years | HR (95% CI) | *P-value* | No. of Cases | Person-years | HR (95% CI) | *P-value* |
| MetS |  |  |  |  |  |  |  |  |  |  |  |  |
| No | 1436 | 3391137 | Reference |  | 1684 | 4612289 | Reference |  | 1531 | 3582806 | Reference |  |
| Yes | 696 | 1658737 | 1.25 (1.14, 1.37) | <0.001 | 909 | 2510866 | 1.31(1.21, 1.42) | <0.001 | 820 | 1925869 | 1.27(1.16, 1.38) | <0.001 |
| Number of MetS | |  |  |  |  |  |  |  |  |  |  |  |
| 0 | 265 | 651584 | Reference |  | 296 | 816438 | Reference |  | 244 | 565849 | Reference |  |
| 1 | 587 | 1361936 | 0.96(0.83, 1.11) | 0.584 | 698 | 1866762 | 0.99(0.86, 1.14) | 0.877 | 634 | 1466198 | 1.03(0.89, 1.20) | 0.696 |
| 2 | 584 | 1377617 | 1.10(0.95, 1.28) | 0.194 | 690 | 1929089 | 1.10(0.96, 1.27) | 0.177 | 653 | 1550759 | 1.20(1.03, 1.39) | 0.019 |
| 3 | 403 | 954269 | 1.18(1.01, 1.39) | 0.038 | 521 | 1444639 | 1.26(1.09, 1.46) | 0.002 | 467 | 1087469 | 1.28(1.09, 1.51) | 0.002 |
| 4 | 225 | 549175 | 1.42(1.18, 1.71) | <0.001 | 294 | 830758 | 1.47(1.25, 1.74) | <0.001 | 272 | 657195 | 1.53(1.28, 1.83) | <0.001 |
| 5 | 68 | 155293 | 1.65(1.26, 2.17) | <0.001 | 94 | 235469 | 1.70(1.34, 2.16) | <0.001 | 81 | 181205 | 1.64(1.27, 2.11) | <0.001 |
| *P* for trend | <0.001 |  |  |  | <0.001 |  |  |  | <0.001 |  |  |  |
| MetS components | |  |  |  |  |  |  |  |  |  |  |  |
| Hypertriglyceridemia | |  |  |  |  |  |  |  |  |  |  |  |
| No | 1178 | 2752858 | Reference |  | 1417 | 3864177 | Reference |  | 1303 | 3040935 | Reference |  |
| Yes | 954 | 2297016 | 1.12 (1.03, 1.22) | 0.011 | 1176 | 3258978 | 1.14 (1.06, 1.24) | <0.001 | 1048 | 2467740 | 1.13 (1.04, 1.23) | 0.004 |
| Reduced HDL cholesterol | | |  |  |  |  |  |  |  |  |  |  |
| No | 1660 | 1660 | Reference |  | 1991 | 5449348 | Reference |  | 1817 | 4263562 | Reference |  |
| Yes | 472 | 472 | 1.24 (1.12, 1.38) | <0.001 | 602 | 1673807 | 1.24 (1.13, 1.36) | <0.001 | 534 | 1245113 | 1.22 (1.1, 1.34) | <0.001 |
| Elevated waist circumference | | |  |  |  |  |  |  |  |  |  |  |
| No | 1297 | 3033759 | Reference |  | 1525 | 4141017 | Reference |  | 1379 | 3180636 | Reference |  |
| Yes | 835 | 2016115 | 1.33 (1.21, 1.45) | <0.001 | 1068 | 2982138 | 1.34 (1.24, 1.45) | <0.001 | 972 | 2328039 | 1.32 (1.21, 1.44) | <0.001 |
| Elevated HbA1c | |  |  |  |  |  |  |  |  |  |  |  |
| No | 1708 | 4107265 | Reference |  | 2031 | 5619855 | Reference |  | 1826 | 4273832 | Reference |  |
| Yes | 424 | 942609 | 1.18 (1.06, 1.32) | 0.003 | 562 | 1503300 | 1.21 (1.10, 1.34) | <0.001 | 525 | 1234843 | 1.19 (1.08, 1.32) | <0.001 |
| Elevated blood pressure | | |  |  |  |  |  |  |  |  |  |  |
| No | 613 | 1459716 | Reference |  | 714 | 1982144 | Reference |  | 596 | 1419482 | Reference |  |
| Yes | 1519 | 3590158 | 1.00(0.91, 1.11) | 0.933 | 1879 | 5141011 | 1.03 (0.94, 1.12) | 0.557 | 1755 | 4089193 | 1.07 (0.97, 1.18) | 0.168 |

Abbreviations: HbA1c, hemoglobin A1c; HDL, high-density lipoprotein; MetS, metabolic syndrome; HR, hazard ratio; 95% CI, 95% confidence interval

Supplemental File 2 Sensitivity analysis of assessing the risk of psoriasis based on metabolic syndrome (MetS) status and genetic susceptibility

|  | Complete missing data of MetS components and covariates | | | | Excl. Participants with a follow-up <2 years | | | | Excl. Fasting time less than 3 hours | | | |
| --- | --- | --- | --- | --- | --- | --- | --- | --- | --- | --- | --- | --- |
|  | No. of Cases | Person-years | HR (95% CI) | *P-value* | No. of Cases | Person-years | HR (95% CI) | *P-value* | No. of Cases | Person-years | HR (95% CI) | *P-value* |
| Low PRS |  |  |  |  |  |  |  |  |  |  |  |  |
| Non MetS | 205 | 487031 | Reference |  | 247 | 663092 | Reference |  | 215 | 491762 | Reference |  |
| MetS | 421 | 1021108 | 1.16 (0.91, 1.49) | 0.235 | 492 | 1343147 | 2.01 (1.73, 2.34) | 0.279 | 444 | 1062575 | 1.13 (0.89, 1.44) | 0.298 |
| Middle PRS | |  |  |  |  |  |  |  |  |  |  |  |
| Non MetS | 203 | 479895 | 1.32 (1.13, 1.54) | <0.001 | 291 | 803411 | 1.65 (1.42, 1.92) | <0.001 | 261 | 603708 | 1.36 (1.17, 1.58) | <0.001 |
| MetS | 402 | 944220 | 1.72 (1.54, 1.93) | 1.69 (1.43, 2.01) | 504 | 1383485 | 1.69 (1.47, 1.94) | <0.001 | 458 | 1086534 | 1.68 (1.43, 1.98) | <0.001 |
| High PRS |  |  |  |  |  |  |  |  |  |  |  |  |
| Non MetS | 810 | 1882998 | 2.08 (1.76, 2.45) | <0.001 | 945 | 2606050 | 1.28 (1.11, 1.47) | <0.001 | 872 | 2028469 | 2.1 (1.78, 2.47) | <0.001 |
| MetS | 91 | 234622 | 2.6 (2.14, 3.16) | <0.001 | 114 | 323970 | 2.88 (2.43, 3.42) | <0.001 | 101 | 235627 | 2.89 (2.41, 3.47) | <0.001 |

Abbreviations: PRS, polygenic risk score; MetS, metabolic syndrome; HR, hazard ratio; 95% CI, 95% confidence interval.

Supplemental File 3 Incidence rate of psoriasis by MetS status and age

|  | 60 and above | | | | Under 60 | | | |
| --- | --- | --- | --- | --- | --- | --- | --- | --- |
|  | No. of Cases | Person-years | HR (95% CI) | *P-value* | No. of Cases | Person-years | HR (95% CI) | *P-value* |
| MetS |  |  |  |  |  |  |  |  |
| No | 910 | 2190174 | Reference |  | 547 | 1293947 | Reference |  |
| Yes | 1103 | 2542198 | 1.17 (1.05, 1.3) | 0.005 | 539 | 1276599 | 1.46 (1.31, 1.62) | <0.001 |
| Number of MetS | |  |  |  |  |  |  |  |
| 0 | 111 | 267266 | Reference |  | 240 | 568029 | Reference |  |
| 1 | 387 | 922625 | 0.85(0.69, 1.05) | 0.129 | 439 | 991324 | 1.05(0.89, 1.23) | 0.571 |
| 2 | 412 | 1000283 | 0.91(0.74, 1.12) | 0.375 | 424 | 982845 | 1.26(1.07, 1.48) | 0.005 |
| 3 | 315 | 743028 | 1.00(0.80, 1.24) | 0.976 | 313 | 740670 | 1.49(1.25, 1.77) | <0.001 |
| 4 | 174 | 425399 | 1.09(0.86, 1.39) | 0.483 | 171 | 420283 | 1.76(1.44, 2.15) | <0.001 |
| 5 | 58 | 125520 | 1.20(0.87, 1.65) | 0.272 | 55 | 115646 | 2.32(1.72, 3.12) | <0.001 |
| *P* for trend | 0.006 |  |  |  | <0.001 |  |  |  |
| MetS components | |  |  |  |  |  |  |  |
| Hypertriglyceridemia | |  |  |  |  |  |  |  |
| No | 795 | 1878459 | Reference |  | 891 | 2083454 | Reference |  |
| Yes | 662 | 1605662 | 1.02(0.92, 1.13) | 0.726 | 751 | 1735343 | 1.3(1.17, 1.44) | <0.001 |
| Reduced HDL cholesterol | | |  |  |  |  |  |  |
| No | 1151 | 2754052 | Reference |  | 1223 | 2834161 | Reference |  |
| Yes | 306 | 730069 | 1.14(1.01, 1.30) | 0.041 | 419 | 984636 | 1.30(1.16, 1.46) | <0.001 |
| Elevated waist circumference | | |  |  |  |  |  |  |
| No | 851 | 2057501 | Reference |  | 977 | 2195903 | Reference |  |
| Yes | 606 | 1426620 | 1.18(1.06, 1.32) | 0.002 | 665 | 1622894 | 1.47(1.33, 1.63) | <0.001 |
| Elevated HbA1c | |  |  |  |  |  |  |  |
| No | 1060 | 2558870 | Reference |  | 1369 | 3201660 | Reference |  |
| Yes | 397 | 925251 | 1.15(1.02, 1.29) | 0.02 | 273 | 617137 | 1.30(1.14, 1.49) | <0.001 |
| Elevated blood pressure | | |  |  |  |  |  |  |
| No | 286 | 690252 | Reference |  | 565 | 1340421 | Reference |  |
| Yes | 1171 | 2793869 | 0.97(0.85, 1.11) | 0.669 | 1077 | 2478376 | 1.10(0.99, 1.22) | 0.070 |

Abbreviations: HbA1c, hemoglobin A1c; HDL, high-density lipoprotein; MetS, metabolic syndrome; HR, hazard ratio; 95% CI, 95% confidence interval.

Supplemental File 4 Incidence rate of psoriasis by age based on MetS status and genetic susceptibility

|  | 60 and above | | | | Under 60 | | | |
| --- | --- | --- | --- | --- | --- | --- | --- | --- |
|  | No. of Cases | Person-years | HR (95% CI) | *P-value* | No. of Cases | Person-years | HR (95% CI) | *P-value* |
| Low PRS |  |  |  |  |  |  |  |  |
| Non MetS | 151 | 358343 | Reference |  | 181 | 424536 | Reference |  |
| MetS | 300 | 704918 | 1.07 (0.8, 1.42) | 0.647 | 58 | 147145 | 1.17 (0.86, 1.59) | 0.31 |
| Middle PRS | |  |  |  |  |  |  |  |
| Non MetS | 305 | 713882 | 1.31 (1.08, 1.58) | 0.005 | 74 | 181359 | 1.95 (1.60, 2.37) | <0.001 |
| MetS | 330 | 768922 | 1.48 (1.21, 1.81) | <0.001 | 622 | 1414933 | 2.06 (1.72, 2.46) | <0.001 |
| High PRS |  |  |  |  |  |  |  |  |
| Non MetS | 249 | 606164 | 1.92 (1.56, 2.36) | <0.001 | 525 | 1264867 | 2.13 (1.76, 2.58) | <0.001 |
| MetS | 136 | 319143 | 2.48 (1.98, 3.12) | <0.001 | 168 | 398706 | 3.49 (2.81, 4.34) | <0.001 |

Abbreviations: PRS, polygenic risk score; MetS, metabolic syndrome; HR, hazard ratio; 95% CI, 95% confidence interval.

Supplemental File 5 Incidence rate of psoriasis by MetS status and gender

|  | Women | | | | Men | | | |
| --- | --- | --- | --- | --- | --- | --- | --- | --- |
|  | No. of Cases | Person-years | HR (95% CI) | *P-value* | No. of Cases | Person-years | HR (95% CI) | *P-value* |
| MetS |  |  |  |  |  |  |  |  |
| No | 1039 | 2437400 | Reference |  | 534 | 1229383 | Reference |  |
| Yes | 974 | 2294972 | 1.36 (1.22, 1.51) | <0.001 | 552 | 1341163 | 1.25 (1.12, 1.39) | <0.001 |
| Number of MetS | |  |  |  |  |  |  |  |
| 0 | 242 | 573822 | Reference |  | 109 | 261473 | Reference |  |
| 1 | 441 | 1024502 | 1.00(0.85, 1.17) | 0.999 | 385 | 889447 | 1.00(0.81, 1.24) | 0.993 |
| 2 | 356 | 839076 | 1.06(0.90, 1.26) | 0.490 | 480 | 1144052 | 1.21(0.98, 1.49) | 0.078 |
| 3 | 305 | 693663 | 1.36(1.14, 1.62) | 0.001 | 323 | 790035 | 1.24(1.00, 1.55) | 0.055 |
| 4 | 171 | 409342 | 1.40(1.15, 1.72) | 0.001 | 174 | 436340 | 1.52(1.19, 1.94) | 0.001 |
| 5 | 58 | 126378 | 1.54(1.14, 2.06) | 0.004 | 55 | 114788 | 1.97(1.42, 2.74) | <0.001 |
| *P* for trend | <0.001 |  |  |  | <0.001 |  |  |  |
| MetS components | |  |  |  |  |  |  |  |
| Hypertriglyceridemia | |  |  |  |  |  |  |  |
| No | 973 | 2289237 | Reference |  | 713 | 1672676 | Reference |  |
| Yes | 600 | 1377546 | 1.21(1.09, 1.34) | <0.001 | 813 | 1963459 | 1.10(1.00, 1.22) | 0.060 |
| Reduced HDL cholesterol | |  |  |  |  |  |  |  |
| No | 1162 | 2697712 | Reference |  | 1212 | 2890501 | Reference |  |
| Yes | 411 | 969071 | 1.2(1.07, 1.35) | 0.002 | 314 | 745634 | 1.28(1.13, 1.45) | <0.001 |
| Elevated waist circumference | | |  |  |  |  |  |  |
| No | 869 | 2012303 | Reference |  | 959 | 2241101 | Reference |  |
| Yes | 704 | 1654480 | 1.36(1.23, 1.5) | <0.001 | 567 | 1395034 | 1.29(1.16, 1.43) | <0.001 |
| Elevated HbA1c | |  |  |  |  |  |  |  |
| No | 1274 | 2978941 | Reference |  | 1155 | 2781589 | Reference |  |
| Yes | 299 | 687842 | 1.12(0.98, 1.27) | 0.100 | 371 | 854546 | 1.28(1.13, 1.45) | <0.001 |
| Elevated blood pressure | | |  |  |  |  |  |  |
| No | 545 | 1302821 | Reference |  | 306 | 727852 | Reference |  |
| Yes | 1028 | 2363962 | 1.01(0.9, 1.12) | 0.923 | 1220 | 2908283 | 1.08(0.95, 1.23) | 0.230 |

Abbreviations: HbA1c, hemoglobin A1c; HDL, high-density lipoprotein; MetS, metabolic syndrome; HR, hazard ratio; 95% CI, 95% confidence interval.

Supplemental File 6 Incidence rate of psoriasis by age based on MetS status and genetic susceptibility

|  | Women | | | | Men | | | |
| --- | --- | --- | --- | --- | --- | --- | --- | --- |
|  | No. of Cases | Person-years | HR (95% CI) | *P-value* | No. of Cases | Person-years | HR (95% CI) | *P-value* |
| Low PRS |  |  |  |  |  |  |  |  |
| Non MetS | 148 | 361936 | Reference |  | 593 | 1353428 | Reference |  |
| MetS | 298 | 722036 | 1.31 (0.99, 1.74) | 0.057 | 73 | 168067 | 0.94 (0.70, 1.28) | 0.709 |
| Middle PRS | |  |  |  |  |  |  |  |
| Non MetS | 295 | 694919 | 1.33 (1.11, 1.59) | 0.002 | 166 | 366397 | 1.33 (1.11, 1.61) | 0.002 |
| MetS | 139 | 315550 | 1.76 (1.44, 2.15) | <0.001 | 554 | 1326372 | 1.63 (1.34, 2.00) | <0.001 |
| High PRS |  |  |  |  |  |  |  |  |
| Non MetS | 281 | 653050 | 2.03 (1.67, 2.48) | <0.001 | 59 | 160437 | 2.03 (1.66, 2.49) | <0.001 |
| MetS | 310 | 723881 | 2.93 (2.34, 3.67) | <0.001 | 183 | 456845 | 2.96 (2.37, 3.7) | <0.001 |

Abbreviations: PRS, polygenic risk score; MetS, metabolic syndrome; HR, hazard ratio; 95% CI, 95% confidence interval.


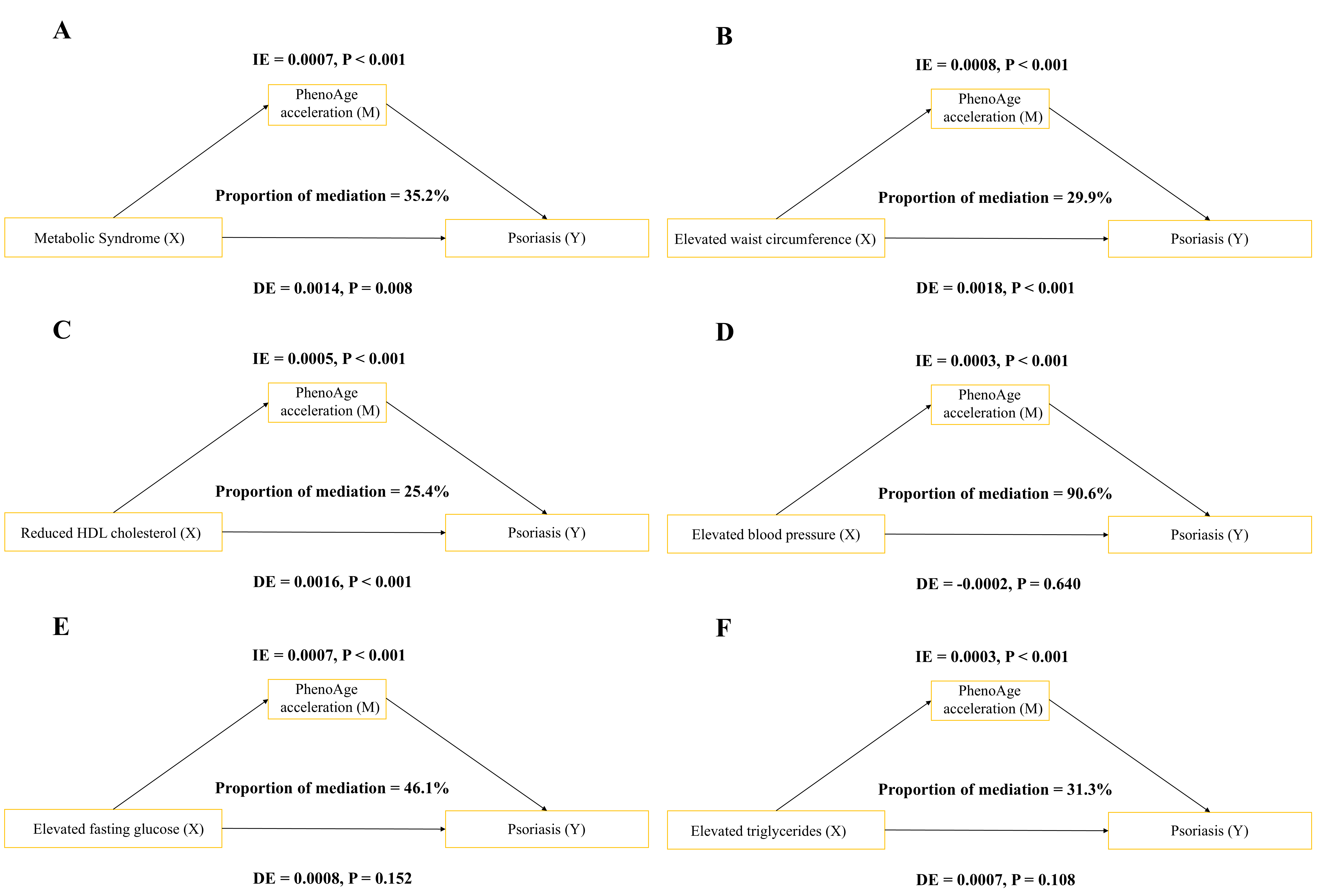


Supplemental figure1 PhenoAgeAccel as a mediator between metabolic syndrome and psoriasis after excluding missing data


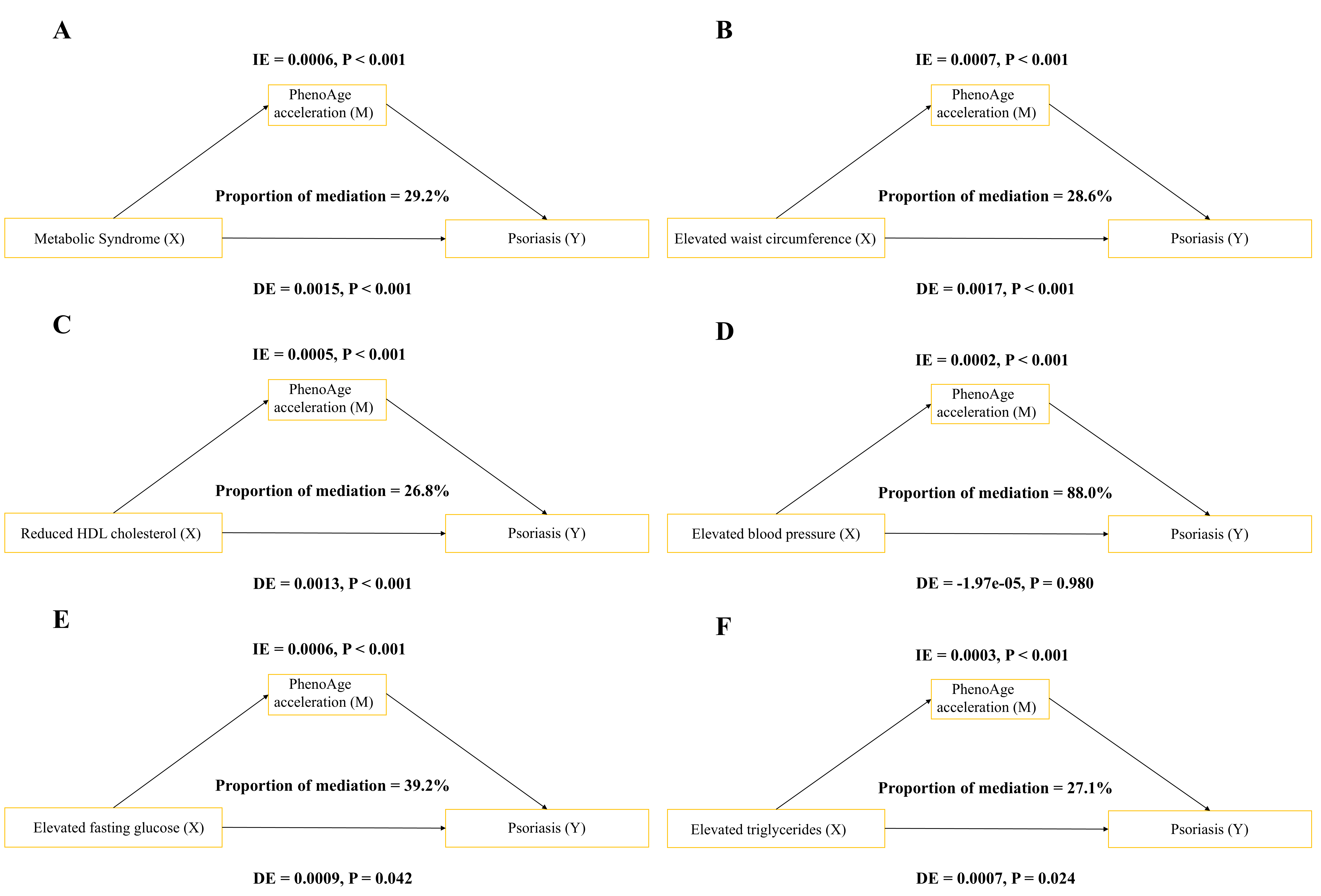


Supplemental figure2 PhenoAgeAccel as a mediator between metabolic syndrome and psoriasis after excluding participants with a follow-up <2 years


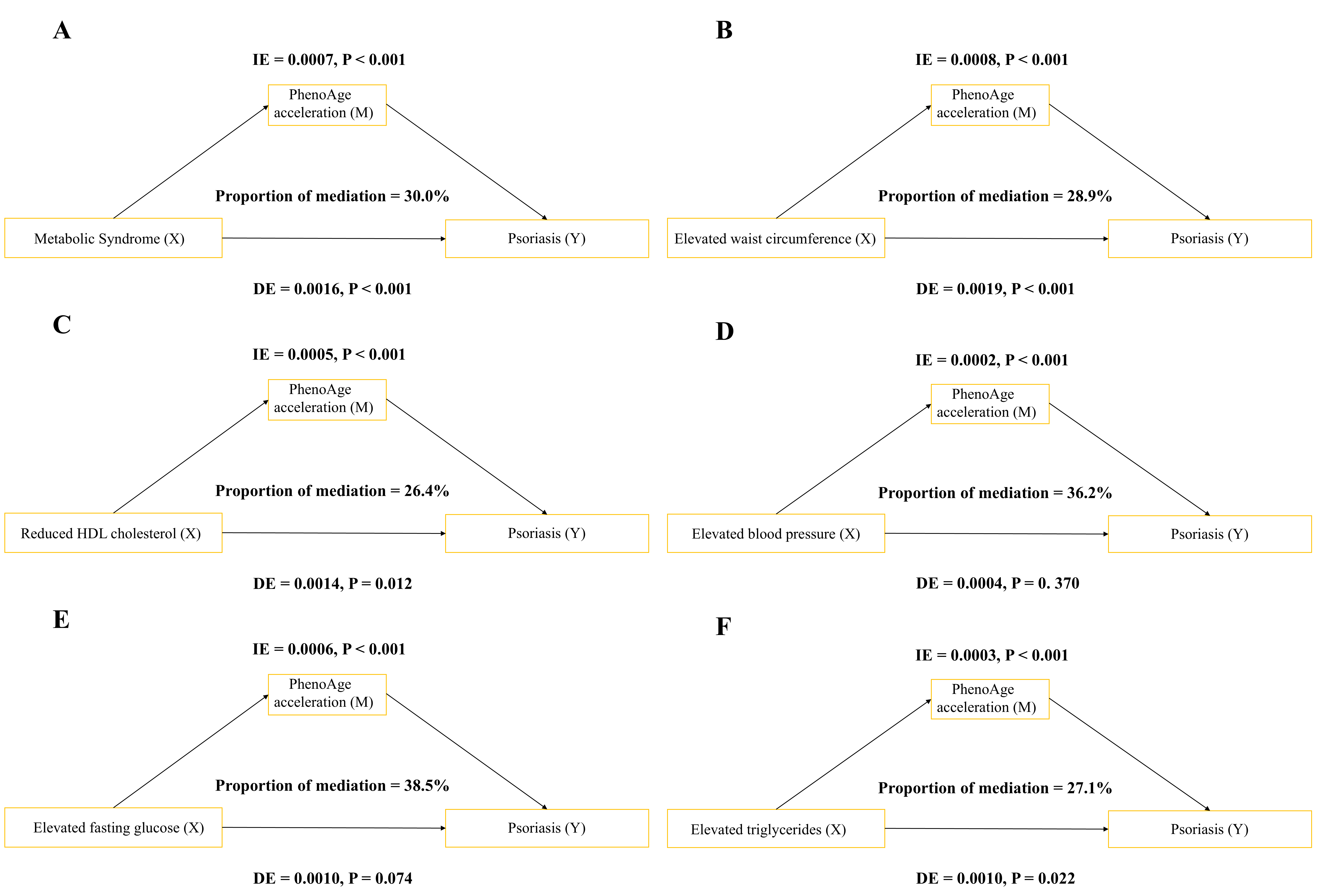


Supplemental figure3 PhenoAgeAccel as a mediator between metabolic syndrome and psoriasis afterexcluding fasting time less than 3 hours
